# Anti-ischemic effect of *Tamarindus indica *L. seed extract against myocardial hypoxic injury

**DOI:** 10.12688/f1000research.126051.1

**Published:** 2022-10-31

**Authors:** Sirirat Surinkaew, Podsawee Mongkolpathumrat, Veeranoot Nissapatorn, Sarawut Kumphune

**Affiliations:** 1 Research Excellence Center for Innovation and Health Products (RECIHP), Walailak University, Nakhon Si Thammarat, 80160, Thailand; 2 School of Allied Health Sciences, Walailak University, Nakhon Si Thammarat, 80160, Thailand; 3 Biomedical Engineering Institute, Chiang Mai University, Chiang Mai, 50200, Thailand

**Keywords:** tamarind seed, hypoxia, antioxidant, anti-inflammatory, anti-apoptosis, H9c2

## Abstract

**Background:** Ischemic heart disease is a leading cause of death in patients with cardiovascular disease. Natural products containing high antioxidant activity have been used as an alternative therapy to improve the living conditions of patients. In this study, we examine the protective effect of tamarind seed (TS) on myocardial hypoxic injury.

**Methods:** The hypoxia model was mimicked by mineral oil overlayed on H9c2 cardiomyoblasts for 4 h. TS extract was pretreated and administered during the hypoxic condition. Radical scavenging activity of TS extract was measured and exhibited very potent antioxidant activities on 2,2-diphenyl-2-picrylhydrazyl (DPPH) and 2,2′-Azino-bis (3-ethylbenzothiazoline-6-sulfonic acid (ABTS) assays.

**Results:** TS extract at a concentration of 10 µg/ml significantly reversed the effect of hypoxia-induced cell death and intracellular reactive oxygen species (ROS) production. We also observed hypoxia-induced over-expression of both inflammatory cytokine mRNA and activation of cellular apoptosis. Pretreatment of TS extract significantly reduced hypoxia-induced HIF-1a and pro-inflammatory cytokine production, IL-1b and IL-6. The Western blot analysis for apoptotic regulatory molecules, caspase 3, caspase 8 and Bax proteins, also showed hypoxia injury reversal by TS extract treatment.

**Conclusions:** The results suggest that the anti-ischemic effect of TS extract protects against hypoxia-induced injury and has potential to be an effective alternative therapy for ischemic heart disease and oxidative-damage related disease.

## Introduction

Ischemic heart disease (IHD) is a heart condition response to deprivation of oxygen and blood supply, ultimately causing heart attack or acute myocardial infarction (AMI). IHD is considered the most prevalent cardiovascular disease and continues to rise. ^1^^,^^2^ Epidemiological data from 2016 to 2019 shows a rise in the number of patients suffering from IHD, which was the leading cause of death in the global population. ^3^ The pathological cause that leads to IHD is the development of atherosclerosis. ^4^ and metabolic risk factors, particularly high BMI, diabetes mellitus, hypertension and high cholesterol, ^1^ leading to plaque buildup, narrowing of arteries and blood flow blockage. The impairment of mitochondrial respiratory chain and oxidation of ferrous heme in the oxymyoglobin complex are the potential sources of reactive oxygen species (ROS) formation upon the onset of ischemia. ^5^^,^^6^ Excessive ROS production with an imbalance of the antioxidant defense system increases inflammation and oxidative stress in IHD patients. ^7^ ROS can also directly injure cell membranes, causing cell death. ^8^ An effective treatment of IHD will thus lead to significant alleviation of strain on the healthcare system and a profound improvement in the quality of life.

Although several kinds of treatment for IHD have been developing, ^9^ the paradigm shift in medical care tends to focus on preventive care. Therefore, early prevention with antioxidant-based therapy is prone to lower the risk of complications from ischemia injury and continues to be an alternative treatment approach. ^10^^,^^11^ A growing number of studies have demonstrated that herbal medication rich in antioxidant properties attenuates oxidative stress, preserves mitochondrial function and exhibits cardioprotective effects. ^12^^,^^13^

*Tamarindus indica* L., commonly known as tamarind, is abundant in India and tropical regions. It has long been used as a spice, snack, food component and traditional medicine. ^14^ As a traditional medicine, tamarind pulp has been used as a laxative, digestive, antioxidant, carminative and antipyretic. ^15^ Tamarind seed (TS) is consequently a commonly discarded and undervalued component of tamarind products. Interestingly, the seeds also exhibit biological and pharmacological effects, as do the other parts of tamarind. The previous reports showed that TS could enhance wound healing and anti-aging, as well as function as an immunomodulatory, renoprotective and anti-diabetic treatment. ^16^^–^^20^ Ethanolic extract of TS exhibits antioxidant properties due to its high content of flavonoid, tannin, polyphenol, anthocyanin and oligomeric proanthocyanidins. ^19^^,^^21^ Ground TS could lower blood glucose, serum cholesterol level, and enhance the storage of glycogen in a hypertensive rat model. ^22^ The possible explainable mechanisms could be its free radical-scavenging activity, antioxidant system homeostasis balancing, and attenuating inflammatory mediators. ^23^^,^^24^

There are intensive bioactivity studies on many parts of *Tamarind indica*; however, the direct effects of TS on cardiomyocytes subjected to ischemic injury have not been reported. Therefore, in the present study, we determine an *in vitro* anti-ischemic effect of TS on cardiac cells subjected to hypoxic injury. Moreover, we examined the effects of TS on Hypoxia-inducible factor-1 alpha (HIF-1α) expression, inflammatory cytokine expression, intracellular ROS production, and cellular apoptosis under hypoxic injury.

## Methods

### Plant material preparation and extraction

Ethical approval for plant use in this study was waived by the Research Institute for Health Sciences, Walailak University, which also approved the research protocol.

*Tamarindus indica* L. fruits were collected in the southern part of Thailand during the peak harvest season from December, 2020 to January, 2021. The seeds were removed and thoroughly washed with tap water, dried in a hot air oven overnight and ground with an electric blender. Then, 50 g of the dried seed powder was extracted in 200 ml of 95% ethanol for seven days at room temperature. The extract solution was filtered and then concentrated under reduced pressure at 40°C using a rotary evaporator, Rotavapor ^®^ R-100 (Buchi Labortechnik AG, Flawil, Switzerland). The sample was air-dried at room temperature to eliminate the remainder of the solvent. ^25^ Crude extract of *Tamarindus indica* L. seeds (TS) was dissolved in 100% dimethyl sulfoxide (DMSO) and stored at 4°C for further experiments.

### DPPH radical scavenging activity of TS extract

An *in vitro* free radical scavenging activity was performed by 2,2-diphenyl-2-picrylhydrazyl (DPPH) (Sigma-Aldrich) scavenging assay. ^26^ Ten millimolar DPPH solution was diluted in absolute methanol and the working reagent of DPPH was adjusted to an optical density of 0.07 ± 0.02 at 517 nm. Ten microliters of different concentrations (0.125–0.4 mg/ml) of the TS extracts and ascorbic acid (used as standard) were thoroughly mixed with 90 μl of freshly prepared DPPH reagent in a 96-well plate. The reaction mixture was incubated in the dark for 30 mins at room temperature. The absorbance was measured at 517 nm with Eon ^TM^ Microplate Spectrophotometer (BioTek Instruments, Inc. Vermont, USA). The measurement was repeated with three individual experiments. The decrease of absorbance determined scavenging activity. The percentage of DPPH radical scavenging activity was calculated from [(A _517_ of control – A _517_ of sample)/A _517_ of control] × 100. IC _50_ value was determined from the percentage inhibition graph, obtained using the *y = mx + c* formula from the slope of the graph.

### ABTS radical scavenging activity of TS extract

The 2,2′-Azino-bis (3-ethylbenzothiazoline-6-sulfonic acid (ABTS) assay is based on decolorization occurring when blue/green ABTS ^•+^ is reduced by antioxidants to ABTS ^•^ (2,2′-Azino-bis (3-ethylbenzothiazoline-6-sulfonic acid) diammonium salt (Sigma-Aldrich). ABTS was dissolved to a concentration of 7 mM in water. ^27^ The radicals of ABTS ^•+^ were generated by reaction of 7 mM ABTS ^•^ and 2.45 mM potassium persulfate at a 1:1 ratio. The mixture was incubated in the dark for 16 to 18 h at room temperature. Then, the ABTS ^•+^ solution was further diluted in absolute methanol until an absorbance value of 734 nm was reached at 0.07 ± 0.02 optical density. The different concentrations (0.125–0.4 mg/ml) of TS extract and ascorbic acid standard were mixed with 90 μl of ABTS ^•+^ working solution in a 96-well plate. The reactions were incubated for 45 mins at room temperature and the absorbance was measured at 734 nm. The experiment was performed in triplicate. The percentage of ABTS ^•+^ scavenging activity was calculated as [(A _734_ of control – A _734_ of sample)/A _734_ of control] × 100. IC _50_ value was determined from the percentage inhibition graph obtained using the *y = mx + c* formula from the slope of the graph.

### Cell culture and hypoxic induction

The rat cardiomyoblast cell line (H9c2) was obtained from ATCC ^®^ CRL-1446 (RRID:CVCL_0286). The cells were cultured in Dulbecco’s Modified Eagle Medium (DMEM, Invitrogen) with 10% fetal bovine serum and 1% penicillin/streptomycin (Thermo Fisher Scientific). Cells were cultured for 5 to 7 days and maintained in 5% CO _2_/95%-humidified air at 37°C.

After cells reached 80% confluence, cells were trypsinized with 0.05% trypsin and plated with a density of 5 × 10 ^3^ cells/well in 96-well plates for 48 h. Cell culture media was replaced with serum free medium before being subjected to hypoxia for 4 h. Hypoxic condition was induced by overlayed mineral oil on the culture media to mimic hypoxic conditions. ^28^^,^^29^ In some experiments, cells were pretreated with TS extract for a 48-h period before and during hypoxic induction.

### Cell viability

Cell viability was determined by 3-(4,5-dimethyl-2-thiazolyl)-2,5-diphenyl-2-H-tetrazolium bromide (MTT) assay. The medium was replaced with 0.5 mg/ml MTT (Invitrogen, Thermo Fisher Scientific) in phosphate buffer saline (PBS) and incubated in the dark for 4 h at 37 °C. The medium was then removed and 100 μl dimethyl sulfoxide (DMSO) was added into each well. The absorbance values were measured at 570 nm using the Eon ^TM^ Microplate Spectrophotometer (BioTek Instruments, Inc., VT, USA). The relative percentage of cell viability was compared with the control group.

### Determination of intracellular ROS production

Cells were seeded in a black-wall 96-well plate at a density of 5 × 10 ^3^ cells/well and maintained in completed DMEM overnight in 5% CO _2_/95%-humidified air at 37°C. The medium was discarded and replaced with condition media containing 25 μM 2′,7′-dichlorofluorescin diacetate (DCFH-DA, Sigma-Aldrich). In the hypoxia group, mineral oil was overlayed on condition media and incubated in 5% CO _2_/95%-humidified air at 37°C for 4 h. In the presence of ROS, the DCHF-DA was rapidly oxidized to highly fluorescent 2′,7′-dichlorofluorescine (DCF). After removing the leftover dye, PBS was added and the intracellular production of ROS was measured by fluorescence intensity with excitation and emission wavelengths of 498 and 522 nm, respectively, with Synergy Mx Microplate Reader (BioTek Instruments Inc., VT, USA).

### RNA extraction and quantitative RT-PCR

To measure hypoxic condition, HIF-1α was determined. GENEzol ^TM^ Reagent (Geneaid, Taiwan) was added directly to 7 × 10 ^4^ cultured cells in a 12-well plate. The cells were lysed by pipetting several times and the sample mixture was incubated for 5 mins at room temperature (RT). Total RNA from the cells was extracted with a GF-1 extraction kit (catalog #GF-TR-100, Vivantis Technologies, Malaysia). The sample was transferred into an RNA Binding Column assembled in a collection tube and centrifuged at 10,000 × g for 1 min then the flow-through was discarded. Wash buffer provided by the kit was then added and centrifuged at 10,000 × g for 1 min. DNase I Digestion Mix provided by the kit was subsequently added into the RNA Binding Column and incubated at RT for 15 mins. Inhibitor Removal Buffer was then added, centrifuged and the flow-through was discarded. The membrane was washed twice with Wash Buffer before the column was placed into a new microcentrifuge tube. Forty microliters of RNase-free water were added directly into the membrane and incubated for 1 min before being centrifuged at 10,000 × g for 1 min. The RNA concentration was determined by NanoDrop One ^C^ (Thermo Fisher Scientific, MA, USA). Complementary DNA synthesis was performed using 0.1 μg of RNA with Viva cDNA Synthesis Kit (catalog #cDSK01-050, Vivantis Technologies, Selangor, Malaysia) following the manufacturer’s protocol.

Quantitative PCR (qPCR) reaction mix was prepared using iTaq™ Universal SYBR ^®^ Green Supermix kit (catalog #1725121, Bio-Rad Laboratories, CA, USA). Ten microliters of iTaq™ Universal SYBR Green Supermix (2X) was mixed with 100 ng of cDNA and 1 μl of 200 mM F + R primers. The total reaction mix volume was adjusted to 20 μl with diethyl pyrocarbonate (DEPC) water. The software StepOnePlus™ Real-Time PCR system (Applied Biosystems, Waltham, MA, USA) was set with a thermal cycle as follows: holding stage 95 °C for 30 s; cycling stage at 95 °C for 15 s; 60 °C for 60 s for 40 cycles; and then melting curve stage at 95 °C for 15 s, 60 °C for 60 s, and 95 °C for 15 s with a temperature increase of 0.3 °C. SYBR Green primers were HIF-1α (Forward primer: 5′ ACACAGAAATGGCCCAGTGAG; Reverse primer: 5′ CACCTTCCACGTTGCTGACTT), ^30^ IL-1β (Forward primer: 5′ CACCTCTCAAGCAGAGCACAG; Reverse primer: GGGTTCCATGGTGAAGTCAAC) and IL-6 (Forward primer: 5′ TCCTACCCCAACTTCCAATGCTC; Reverse primer: TTGGATGGTCTTGGTCCTTAGCC). ^31^ Measurements were performed in triplicates and relative gene expression levels were normalized to endogenous reference gene, GAPDH (Forward primer: 5′ TTCCTACCCCCAATGTATCCG; Reverse primer: 5′ CATGAGGTCCACCACCCTGTT). ^32^ Relative fold change in expression was calculated using the 2 ^-∆∆Ct^ method.

### Western blot analysis

Cells were seeded in a six-well plate at a density of 1 × 10 ^5^ cells/well. At the end of each experiment, the culture plate was placed on ice and then the media was removed. Cells were washed with ice-cold PBS and proteins were collected with 2X Laemmli Sample Buffer (catalog #161-0737, Bio-Rad Laboratories, CA, USA) containing 2-mercaptoethanol and homogenized with insulin syringes. The lysates in 2X Laemmli buffer were denatured at 95°C for 5 mins in a heating box. The cellular protein was separated on 12% SDS-polyacrylamide gel at 90 V for 10 mins followed by 100 V for ~90 mins and then was transferred to polyvinylidene fluoride (PVDF) membranes (100 V; 100 mins). The membranes were blocked with 5% nonfat dry milk in Tris-buffered saline (pH 7.4) containing 0.1% Tween-20 (TBST) for 1 h, followed by incubation with primary antibodies, at the dilution of 1:1,000, cleaved caspase-3 rabbit polyclonal antibody (catalog #9661, RRID:AB_2341188, Cell Signaling Technology, MA, USA), cleaved caspase-8 rabbit monoclonal antibody (catalog #9496, RRID:AB_561381, Cell Signaling Technology, MA, USA), Bax rabbit polyclonal antibody (catalog #sc-493, RRID:AB_2227995, Santa Cruz Biotechnology, Inc., TX, USA) and Bcl-2 rabbit polyclonal antibody (catalog #sc-492, RRID:AB_2064290 , Santa Cruz Biotechnology, Inc., TX, USA) in 1% nonfat dry milk in TBST at 4°C overnight. Membranes were washed and incubated with 1:5,000 dilution goat anti-rabbit IgG secondary antibody, horseradish peroxidase (HRP)-conjugated (catalog #AP132P, Merck & Co., NJ, USA) at room temperature for 1 h. Signals were visualized by Affinity ^®^ Western blot ECL kit (Affinity Biosciences, OH, USA). The digital images of chemiluminescent Western blots were obtained from ImageQuant ^TM^ LAS 500 (GE Healthcare, Life Sciences, NJ, USA). Results were expressed relative to β-actin (Santa Cruz Biotechnology) expression bands on the same samples. The protein quantifications were performed using ImageJ (Version 1.53) (RRID:SCR_003070).

### Statistical analysis

All data are expressed in mean ± SEM. Statistical comparisons between each time point and a single control group were performed with one-way ANOVA followed by Tukey’s multiple comparisons test. The comparisons between two groups of studies were performed with unpaired Student’s t tests. Statistical analyses were performed using GraphPad Prism (Version 9.0). All data analyses performed using this software have been included in the manuscript. ^33^ A p-value of <0.05 was considered statistically significant. R programming language is an open-access alternative that can perform functions equivalent to GraphPad Prism software.

## Results

### Hypoxia reduced cardiac cell viability

**Figure 1.**
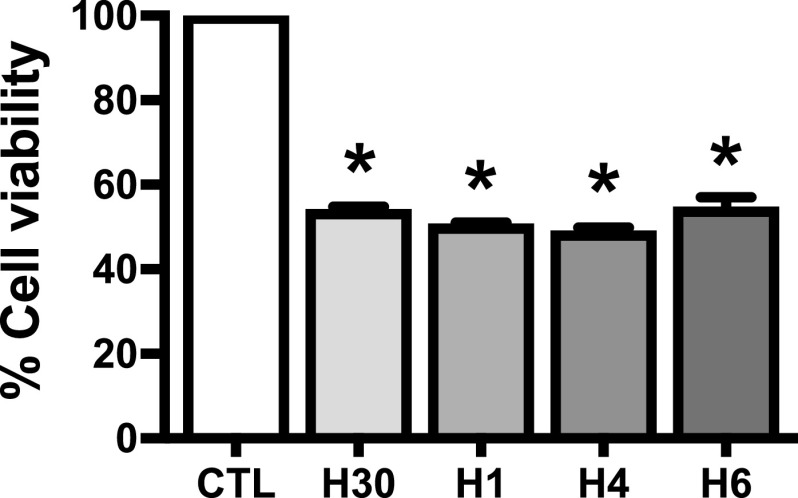
Duration of hypoxia at different time points: 30 mins, 1 h, 4 h or 6 h on cell viability compared with control (CTL). * p < 0.0001 vs. CTL, one-way ANOVA followed by Tukey’s multiple comparisons test.

To optimize timing of hypoxia-induced cell injury, H9c2 cells were subjected to hypoxic conditions for 30 min, 1 h, 4 h or 6 h. Cell viability was determined by MTT assay. Under hypoxic condition, cell viability was significantly decreased to 54 ± 0.6%, 50 ± 0.7%, 49 ± 1.2% and 54 ± 2.7% at 30 min, 1 h, 4 h or 6 h, respectively, compared with the control ( Figure 1: see also *Underlying data* Fig 1 ^33^).

### Radical scavenging activity of TS extract

**Figure 2.**
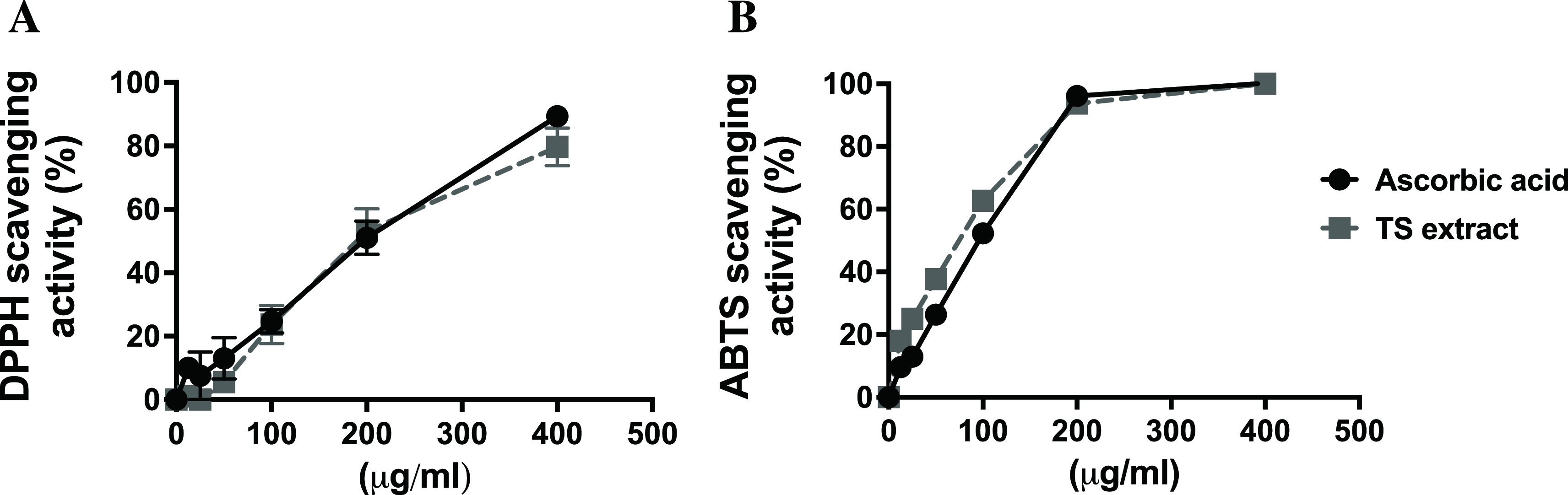
DPPH and ABTS radical scavenging activity of tamarind seed (TS) extract and ascorbic acid (standard). Different concentrations (0.125 – 0.4 mg/ml) of TS extract or ascorbic acid were performed by DPPH (A) and ABTS (B) assay.

Antioxidant activity of TS extract was performed by DPPH and ABTS assays. The crude extract of TS showed potent antioxidant activities obtained from both assays. The DPPH scavenging activity of TS extract displayed 79 ± 5.8% at 0.4 mg/ml and 24 ± 6.1% at 0.1 mg/ml. Similarly, the DPPH radical scavenging activity of ascorbic acid was 89 ± 1.8% at 0.4 mg/ml and 24 ± 3.6% at 0.1 mg/ml ( Figure 2A: see also *Underlying data* Fig 2A ^33^). Moreover, the scavenging activity of ABTS ^+^ radicals of TS extract was observed as a potential antioxidant property; TS extract displayed 100 ± 0.1% scavenging activity at 0.4 mg/ml and 63 ± 2.3% at 0.1 mg/ml, while ascorbic acid served as the standard antioxidant, showing maximum scavenging activity of 100 ± 0.1% at 0.4 mg/ml and 54 ± 2.0% at 0.1 mg/ml ( Figure 2B: see also *Underlying data* Fig 2B ^33^). The IC _50_ values of DPPH free radical scavenging and ABTS radical scavenging were 236 μg/ml and 82 μg/ml, respectively, for TS extract.

### Cytotoxicity of TS extract

**Figure 3.**
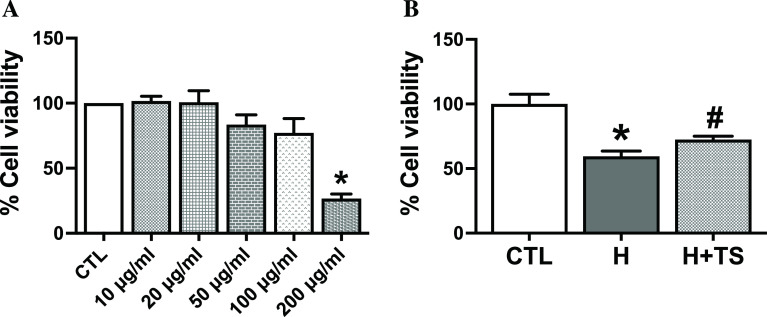
Tamarind seed (TS) induced cytotoxicity was measured by MTT assay. (A) The H9c2 cells were incubated with TS crude extract at different concentrations (10 μg/ml to 200 μg/ml) for 48 h, * p = 0.0272, ** p < 0.0001 vs. control (CTL); (B) TS extract at a concentration of 10 μg/ml increased cell viable in 4 h hypoxia-induced cell death. * p = 0.008 vs. CTL, # p = 0.0151 vs. hypoxia (H).

To determine the optimal concentration of TS on H9c2 cells that does not cause cellular injury, H9c2 was treated with different concentrations of TS crude extract, varying from 10 μg/ml to 200 μg/ml, for 48 h. The cytotoxicity of TS was measured with MTT assay. The results showed that TS extract at concentrations of 10 μg/ml, 20 μg/ml and 50 μg/ml did not affect cell viability when compared with the untreated control group, 101.3 ± 3.6%, 100.9 ± 8.8% and 83.5 ± 4.3%, respectively. However, TS extract at 100 μg/ml and 200 μg/ml significantly reduced cell viability by 76.8 ± 6.4% (p = 0.0272) and 26.2 ± 2.1% (p <0.0001), respectively, compared with the untreated control group ( Figure 3A: see also *Underlying data* Fig 3A ^33^).

### TS extract reduced hypoxia-induced cardiac cell death

To examine the effect of TS extract treatment on cell viability under hypoxia, H9c2 cells were pretreated with TS extract at a concentration of 10 μg/ml for 48 h before being subjected to hypoxia for 4 h. The results showed that hypoxic condition significantly reduced cell viability by MTT assay to 60 ± 4.2%, compared with control (p = 0.0008). Pretreatment with 10 μg/ml of TS extract increased cell viability to 72 ± 2.5%, compared with CTL (p = 0.0151). Therefore, TS extract increased cell viability by 21% compared with hypoxia-induced cell injury ( Figure 3B: see also *Underlying data* Fig 3B ^33^).

### Treatment of TS extract reduced intracellular ROS production

**Figure 4.**
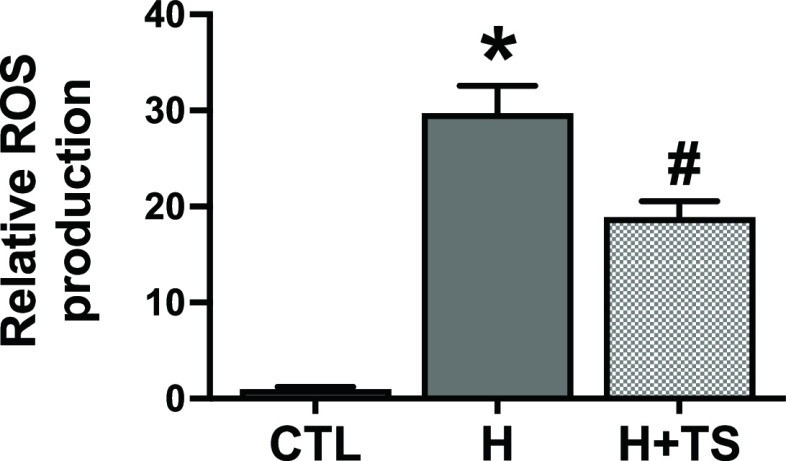
Tamarind seed (TS) extract reduced ROS production. Pretreatment with 10 μg/ml TS extract significantly decreased ROS production. * p < 0.0001 vs. control (CTL), # p = 0.0136 vs. hypoxia (H).

In order to investigate whether TS extract could reduce intracellular ROS production, H9c2 cells were cultured under hypoxic condition for 4 h and measured with DCFH-DA. It was shown that hypoxic condition significantly increased relative ROS production to 29.7 ± 3.4%, compared with CTL (p <0.0001, Figure 4). Pretreatment of 10 μg/ml of TS extract with an additional treatment during hypoxia significantly reduced relative intracellular ROS production to 18.9 ± 2.0 compared with hypoxic group (p = 0.0136, Figure 4: see also *Underlying data* Fig 4 ^33^).

### Treatment of TS extract downregulated HIF-1α and inflammatory cytokines expression

**Figure 5.**
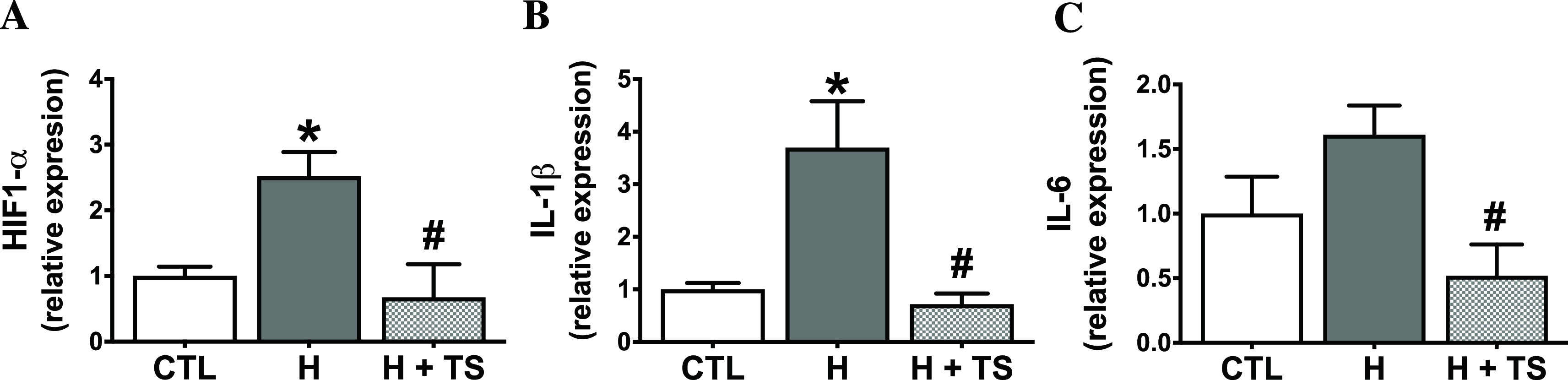
Effects of tamarind seed (TS) extract pretreatment on HIF-1α, IL-1β and IL-6 mRNA expressions. (A) TS extract attenuated HIF-1α mRNA expression and (B) reversed mRNA levels of inflammatory cytokines IL-1β and (C) IL-6 under hypoxic condition. n = 4/group. HIF-1α, * p = 0.0421; IL-1β, * p = 0.0144 vs. control (CTL). HIF-1α, # p = 0.0160; IL-1β, # p = 0.0081; IL-6, # p = 0.0330 vs. hypoxia (H).

The key transcription factor, HIF-1α, is a response to hypoxic injury. The effects of TS extract-mediated cellular inflammation under hypoxic stimulation on HIF-1α mRNA expression were measured. The effects are shown as fold-changes relative to the control (see also *Underlying data* Fig 5A–C ^33^). The results showed that hypoxic condition significantly increased HIF-1α expression by 2.5 ± 0.37 when compared with control (p = 0.0421, Figure 5A), confirming a hypoxic response in H9c2 cells. Treatment with TS extract significantly reduced HIF-1α expression by 3.7-fold, compared with hypoxia group (p = 0.0160, Figure 5A).

To determine whether TS extract mediates inflammation, the mRNA expression of inflammatory cytokines, IL-1β and IL-6, were measured. Under hypoxic condition, the mRNA expressions of IL-1β and IL-6 increased by 3.7 ± 0.89 and 1.6 ± 0.23, respectively, compared with the control ( Figure 5B, C). Most importantly, pretreatment with TS extract significantly attenuated mRNA expression of IL-1β and IL-6 by 5.2- and 3.1-fold, compared with hypoxia, with nearly complete reversal of changes ( Figure 5B, C: see also *Underlying data* Fig 5A–C and supplementary file S1–S7 ^33^). Overall, these results indicate that TS extract protected against hypoxia-induced cytokine production.

### TS extract reduced hypoxia-induced apoptotic regulatory protein level

**Figure 6.**
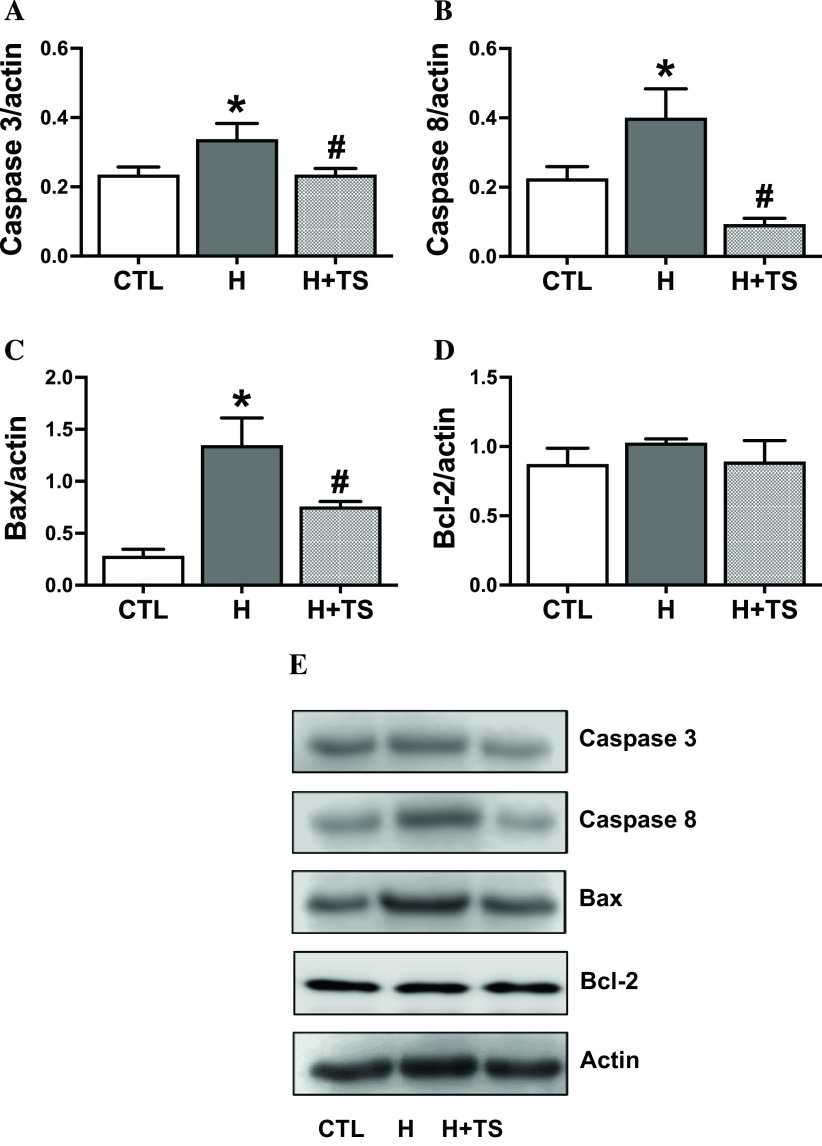
Tamarind seed (TS) extract inhibits hypoxia-induced apoptosis in H9c2 cells. (A-D) Quantitative protein expressions of cleaved-caspase 3, cleaved-caspase 8, Bax and Bcl-2 from H9c2 stimulated with hypoxic condition with or without TS extract (10 μg/ml). n = 4-6/group. Caspase3, * p = 0.0080; Caspase 8, * p = 0.0374; Bax, * p = 0.0003 vs. control (CTL). Caspase3, # p = 0.0206; Caspase 8, # p = 0.0024; Bax, # p = 0.0140 vs. hypoxia (H). (E) Representative immunoblot of cleaved-caspase 3, cleaved-caspase 8, Bax, Bcl-2 and β-actin from H9c2 stimulated with hypoxic condition.

To assess the effect of TS extract on H9c2 cells apoptosis under hypoxic condition, TS extract at a concentration of 10 μg/ml was pretreated for 48 h prior to hypoxia. The levels of apoptotic regulatory protein cleaved-caspase 3, cleaved-caspase 8 and Bax were assessed by Western blot. The representative immunoblots were normalized to actin. The results showed that hypoxic condition significantly increased the level of cleaved-caspase 3, cleaved-caspase 8 and Bax to 0.34 ± 0.04, 0.40 ± 0.08 and 1.35 ± 0.19, respectively, compared with the control ( Figure 6A– C: see also *Underlying data* Fig 6A–6C ^33^). Interestingly, TS extract was markedly decreased in cleaved-caspase 3, cleaved-caspase 8 and Bax levels by 1.4-fold, 4.3-fold and 1.8-fold, respectively, compared with hypoxic condition. However, the level of Bcl-2 protein expression was not different between groups ( Figure 6D– E: see also *Underlying data* Fig 6D–6E and supplementary file S8 ^33^). Our results suggest that TS extract plays an important role in inhibiting cellular apoptosis associated with hypoxic-promoted cell death.

## Discussion

In the present study, we demonstrated for the first time an *in vitro* anti-ischemic effect of tamarind seed (TS) extract on hypoxia-induced cardiac injury by increasing cell viability, reducing ROS accumulation, and lowering HIF-1α mRNA expression. Furthermore, TS extract could reduce inflammatory cytokine IL-1β and IL-6 expression and reduce apoptotic regulatory protein level by decreasing caspase 3, caspase 8 and Bax expression.

Hydroalcoholic TS extract showed a higher amount of antioxidant activity compared with acetone or water-based extraction. ^23^ In the present study, ethanolic extraction of TS showed strong antioxidant activity, similar to ascorbic acid, which was used as a standard, measured by DPPH (2,2-diphenyl-1-picrylhydrazyl) and ABTS free radical scavenging assay. The antioxidant property of TS ethanol extract was confirmed by Luengthanaphol *et al*, 2004. ^34^ The quantitative analysis of phenolic compounds of *Tamarindus indica* seed by HPLC analytical method displayed a high content of antioxidant capacity. ^21^ It was observed that methanolic extracts of TS consist of oligomeric procyanidin tetramer, procyanidin hexamer, procyanidin trimer, procyanidin pentamer, procyanidin B2 and (-)-epicatechin. ^21^ TS-coated extract has also been reported to contain other phenolic antioxidants, such as 2-hydroxy-3’,4’-dihydroxyacetophenone, methyl 3,4-dihydroxybenzoate, 3,4-dihydroxyphenyl acetate, catechin, caffeic acid, ferulic acid, chloramphenicol, myricetin, morin, quercetin, apigenin and kaempferol. ^35^^,^^36^ The polyphenolic compound with the highest content in the methanal seed extract was procyanidin B2, followed by caffeic acid and myricetin. ^36^ As an antioxidant, procyanidin B2 displayed antioxidant properties by inhibiting DNA damage. ^37^ Direct evidence of TS extract protecting cardiomyocytes under oxidative condition has not been previously reported. However, the procyanidin profiles detected in TS are similar to those found in berry, cocoa and dark chocolate and can thus be utilized as an alternative therapy for various cardiovascular diseases, such as angina, hypertension, hyperlipidemia, arrhythmia and congestive heart failure. ^38^^–^^42^

During hypoxia, mitochondria play a role as an O _2_ sensor by increasing the production of ROS, ^43^ which could induce mitochondrial damage, release of cytochrome c, modulate downstream apoptotic cascade through pro-apoptotic factors and subsequently lead to cellular apoptosis. ROS generated from mitochondria can also regulate downstream signaling pathways, including HIF-1α. In cardiac disease, HIF-1α plays a key role in the regulation of multiple genes involved in the maintenance of oxygen homeostasis at a low oxygen level, regulating cell survival and differentiation. The increase of HIF-1α expression is an adaptive response of the body to myocardial ischemia and hypoxia. HIF-1α can increase blood oxygen capacity by regulating angiogenesis, vascular remodeling, regulation glucose metabolism and redox balance and improving oxygen utilization. ^44^ On the other hand, a previous study found that hypoxia can induce inflammation and apoptosis via the nitric oxide (NO)-mediated pathway in cardiomyoblast cells. ^45^ NO is a potent mediator of cellular damage, which can react with superoxide anion to produce peroxynitrite, which, in turn, can interact with DNA, proteins and lipids, leading to oxidative injury and cellular apoptosis. ^46^ Antioxidant effects of TS have been shown to reduce lipid peroxidation ^47^ and inhibit NO production, ^48^ which may result from nitric oxide radical scavenging activity, superoxide anion and hydroxyl ion scavenging activity. ^17^ Here, we showed that hypoxia increased cellular injury involved in the regulation of HIF-1α gene expression, which was attenuated by the scavenging activity of TS extract. TS extract may also decrease metabolic stress induced by hypoxia and ultimately attenuate cellular apoptosis. The direct effect of TS extract on preserving mitochondria metabolism and functions should be further investigated.

It has been reported that TS extract administered in experimental arthritic rat model efficiently maintained the homeostasis of the antioxidant system and significantly reduced serum levels of tumor necrosis factor-α (TNF-α), interleukin (IL)-1β, IL-6, IL-23 and cyclooxygenase-2. ^24^ In this study’s results, we demonstrated that phenolic flavonoids from seed extract reduced ROS levels, and in turn, reduced the production of pro-inflammatory cytokine mediators, IL-1β and IL-6, following exposure to acute hypoxia compared with the control group. The overexpression of caspase 3, caspase 8 and Bax protein expression was reversed by TS extract, which indicated that TS extract can protect against inflammation induced by hypoxia, subsequently preventing apoptosis. The cardioprotective effects of TS may directly scavenge ROS *under oxidative stress, thereby protecting from inflammatory response and cell death.* Overall, TS was observed as a great potential extract to directly reverse myocardial hypoxia-mediated inflammation and cellular apoptosis. TS extract may also reduce inflammation in other diseases, such as inflammatory cardiomyopathy, etc., which should be further studied as an alternative choice.

There are some limitations on the anti-ischemic effect of TS against hypoxic injury, which need to be clarified. During hypoxia, the imbalance between free radical production and elimination can occur. ^49^ According to this study, we showed that TS extract has potential antioxidant activity similar to ascorbic acid, which was supported by other studies. ^19^^,^^50^ Radical scavenging activities of TS extract could directly reduce intracellular ROS accumulation under hypoxic condition in an *in vitro* model. However, antioxidant defense systems, such as glutathione peroxidase, peroxiredoxin, total antioxidant activities, as well as associated transcriptional factors, should be further investigated. We also demonstrated strong evidence of pretreatment of TS extract attenuating the effects of hypoxia induced-inflammatory cytokines and apoptosis, which is likely due to its anti-oxidant and anti-inflammatory properties, as previously reported. ^48^^,^^50^^,^^51^ The underlying mechanism of TS in preventing hypoxic injury remains to be clarified. The p38 MAPK, involving cell differentiation, survival and apoptosis, plays a crucial role in cardiac ischemic response and is known to be regulated by HIF-1α. ^52^^,^^53^ However, the signaling pathway of p38 MAPK regulating HIF-1α through ROS scavenging of TS extract needs to be further elucidated. Furthermore, in order to study the systemic effects of TS extract, including long term treatment, different time point of administration (such as at the onset of ischemia or at the beginning of reperfusion), and the effect of isolated TS compounds, as well as their combinations, a myocardial infarction experimental animal model, should be further performed and its associated mechanisms (such as p38 MAPK) need to be further investigated.

## Conclusions

In the present study, we demonstrated that the anti-inflammatory and anti-apoptotic activity of TS extract protects against hypoxia-induced cardiac cell injury. These beneficial effects may come from the potent antioxidant activity of TS extract, which may be utilized as a natural antioxidant product to reduce the risk of myocardial ischemia.

## Data Availability

Figshare: Underlying data for ‘Anti-ischemic effect of *Tamarindus indica* L. seed extract against myocardial hypoxic injury’, https://doi.org/10.6084/m9.figshare.21066028. ^33^ This project contains the following underlying data:
• Data file 1: Fig 1_Absorbance value of duration of hypoxia.csv • Data file 2: Fig 2A_Absorbance value of DPPH.csv • Data file 3: Fig 2B_Absorbance value of ABTS.csv • Data file 4: Fig 3A_Absorbance value of cytotoxicity.csv • Data file 5: Fig 3B_Absorbance value of cell viability.csv • Data file 6: Fig 4_Fluorescent intensity of ROS production.csv • Data file 7: Fig 5A-C_Relative mRNA expression of HIF-1alpha, IL-1B and IL-6.csv • Data file 8: Fig 6A_Relative protein expression of Caspase3_actin.csv • Data file 9: Fig 6B_Relative protein expression of Caspase8_actin.csv • Data file 10: Fig 6C_Relative protein expression of Bax_actin.csv • Data file 11: Fig 6D_Relative protein expression of Bcl-2_actin.csv • Supplementary file 1: S1_RNA measurement.csv • Supplementary file 2: S2_Raw Ct_GAPDH_HIF-1alpha.csv • Supplementary file 3: S3_Raw Ct_IL1B_IL-6.csv • Supplementary file 4: S4_Raw data quantification_GAPDH_HIF-1alpha.csv • Supplementary file 5: S5_Raw data quantification_IL-1B_IL-6.csv • Supplementary file 6: S6_Amplification data_GAPDH_HIF-1alpha.csv • Supplementary file 7: S7_Amplification data_IL-1B_IL-6.csv • Supplementary file 8: S8_Fig 6E_Original blot.pptx • Supplementary file 9: S9_GraphPad Prism of all data and statistical analysis Data are available under the terms of the Creative Commons Attribution 4.0 International license (CC-BY 4.0)
