# Supplementary material for: Anti-ischemic effect of Tamarindus indica L. seed extract against myocardial hypoxic injury
Source: F1000Res. 2022 Oct 31;11:1235. [Version 1] doi: 10.12688/f1000research.126051.1 (PMC11046188; doi:10.12688/f1000research.126051.1)
Supplement: Peer review history

# Reviewer report: Reviewer response for version 1

**DOI:** 10.5256/f1000research.138426.r253353

**Published:** 2024-04-25

**Authors:** Siva Ramamoorthy

**Affiliations:** 1 Vellore Institute of Technology, Vellore, Tamil Nadu, India

Here are the limitations that I have seen in the paper.

1. TS extract has a potential antioxidant activity similar to ascorbic acid. Radical scavenging activities of TS extract could directly reduce intracellular ROS accumulation under hypoxic conditions in an in vitro model. However, antioxidant defense systems, such as glutathione peroxidase, peroxiredoxin, total antioxidant activities, as well as associated transcriptional factors, should be further investigated. 
2. The authors have demonstrated strong evidence of pretreatment of TS extract attenuating the effects of hypoxia-induced-inflammatory cytokines and apoptosis, which is likely due to its antioxidant and anti-inflammatory properties. The underlying mechanism of TS in preventing hypoxic injury remains to be clarified. 
3. The p38 MAPK, involving cell differentiation, survival, and apoptosis, plays a crucial role in cardiac ischemic response and is known to be regulated by HIF-1α. However, the signaling pathway of p38 MAPK regulating HIF-1α through ROS scavenging of TS extract needs to be further elucidated. 
4. The systemic effects of TS extract, including long-term treatment, different time points of administration (such as at the onset of ischemia or the beginning of reperfusion), and the effect of isolated TS compounds, as well as their combinations in a myocardial infarction experimental animal model should be further performed and its associated mechanisms (such as p38 MAPK) need to be further investigated.

Is the work clearly and accurately presented and does it cite the current literature?

Yes

If applicable, is the statistical analysis and its interpretation appropriate?

Yes

Are all the source data underlying the results available to ensure full reproducibility?

Yes

Is the study design appropriate and is the work technically sound?

Yes

Are the conclusions drawn adequately supported by the results?

Yes

Are sufficient details of methods and analysis provided to allow replication by others?

Yes

Reviewer Expertise:

Natural Products, pigments, cancer biology

I confirm that I have read this submission and believe that I have an appropriate level of expertise to confirm that it is of an acceptable scientific standard.

# Reviewer report: Reviewer response for version 1

**DOI:** 10.5256/f1000research.138426.r253348

**Published:** 2024-04-25

**Authors:** Christophe Wiart

**Affiliations:** 1 Universiti Sabah, Malaysia, Malaysia

This is a well-written paper. The topic is timely. Protocol and methods are okay. The results are okay. I feel that the authors need to explain why no positive standard was used for the cytotoxicity test as well as the hypoxia test. I also feel that the authors could write a lot more about the traditional use of the plant in Thailand and why they chose the seeds only and why not the whole fruit or the pulp. Editing is excellent.

Is the work clearly and accurately presented and does it cite the current literature?

Yes

If applicable, is the statistical analysis and its interpretation appropriate?

Yes

Are all the source data underlying the results available to ensure full reproducibility?

Yes

Is the study design appropriate and is the work technically sound?

Yes

Are the conclusions drawn adequately supported by the results?

Yes

Are sufficient details of methods and analysis provided to allow replication by others?

Yes

Reviewer Expertise:

Natural products, ethnopharmacology, pharmacy

I confirm that I have read this submission and believe that I have an appropriate level of expertise to confirm that it is of an acceptable scientific standard.
